# Early performance of a decentralised, primary-care hepatitis C programme in Cambodia: a retrospective programme evaluation, 2024

**DOI:** 10.1016/j.lanwpc.2025.101758

**Published:** 2025-11-26

**Authors:** Chansovannara Soputhy, Florian Girond, Samley Keo, Kolveasna Kim, Luis Sagaon-Teyssier, Capucine Penicaud, Sovann Ly, Emilie Mosnier

**Affiliations:** a Aix Marseille Univ, Inserm, IRD, SESSTIM, Sciences Economiques & Sociales de la Santé & Traitement de l'Information Médicale, ISSPAM, Marseille, France; b Communicable Disease Control Department, Ministry of Health, Phnom Penh, Cambodia; c Calmette Hospital, Phnom Penh, Cambodia; d University of Health Sciences, Phnom Penh, Cambodia; e Health Management Support Team (HMST), France; f Infectious and Tropical Diseases Unit, University Hospital of La Réunion, La Réunion, France

**Keywords:** Hepatitis C virus, Elimination, Screening, Direct-acting antivirals, Decentralisation, Cambodia

## Abstract

**Background:**

Real-world evidence on decentralised, primary-care delivery for hepatitis C virus (HCV) in the Western Pacific is limited. We evaluated Cambodia's national, primary care–led HCV programme in 2024.

**Methods:**

We analysed facility-level data from 256 health facilities in 15 operational districts to assess six HCV cascade steps: screening, anti-HCV positivity, RNA testing, viraemia, treatment initiation, and treatment completion. Design-based survey estimators were used to estimate proportions with 95% confidence intervals (CIs). To account for multi-stage design (clustering within operational districts), design-based generalised linear models were utilised to assess the factors associated with viraemia, treatment initiation and completion.

**Findings:**

HCV testing coverage among adults (≥18 years; denominator 2,196,351) was 3·5% (95% CI 2·5–4·4). Of 76,512 adults tested, 3213 (4·2%; 95% CI 3·1–5·6) were anti-HCV-positive; 2628/3213 (81·8%) received RNA testing, and 1446/2628 (55·0%; 95% CI 49·5–60·3) were viraemic (1·9% of all tested). Among RNA-positive individuals, 1345/1446 (93·0%) initiated direct-acting antivirals and 1289/1345 (95·8%) completed treatment. Viraemia was higher among men (adjusted odds ratio [aOR] 1·40; 95% CI 1·05–1·86), varied by province (Takeo aOR 2·79, Kampong Cham aOR 2·11 versus Battambang), and elevated in October–December (Q4; aOR 1·79) versus January–March. Treatment initiation and completion surpassed 90% across facilities. The principal gap was confirmatory testing (18·2% of anti-HCV-positive individuals lacked RNA testing).

**Interpretation:**

A decentralised, primary-care model achieved high linkage and treatment completion in the first year. Closing the confirmatory testing gap (reflex RNA/core antigen from same encounter), prioritising low-coverage/high-burden districts, and establishing patient-level linkage to capture sustained virologic response at week 12 are priorities to accelerate elimination.

**Funding:**

ANRS MIE (ANRS0689b).

**Research in context**

**Evidence before this study**

In 2016, WHO set hepatitis C virus (HCV) elimination targets for 2030. Direct-acting antivirals (DAAs) transformed HCV care and catalysed national programmes—ranging from decentralised primary-care delivery to telemedicine—yet only about eleven countries are currently on track to meet the targets. Cambodia's general-population HCV prevalence was approximately 1·2% in 2022, higher than neighbouring countries. In 2023, Cambodia launched a national elimination programme adopting a decentralised model informed by Médecins Sans Frontières pilots, initially across 15 operational districts in six provinces. We searched PubMed narratively using (“Hepatitis C” OR HCV) AND (“health services accessibility” OR “primary health care”) AND (“programme evaluation” OR evaluation) AND (systematic), without date or language limits. We identified seven systematic reviews, most reporting that decentralised models can improve screening, linkage to care, and treatment outcomes. However, programme-wide evaluations using routine data from multi-province roll-outs in resource-limited settings remain scarce, especially on early cascade performance and equity.

**Added value of this study**

To our knowledge, this is the first report of outcomes from the National Viral Hepatitis Elimination Programme operationalised at 256 healthcare facilities, streamlining the pathway from screening to treatment completion in about four visits in Cambodia. In 2024, among 76,512 adults tested, 4·2% were anti-HCV positive; 81·8% of seropositive individuals received RNA testing; 55·0% of those tested were viraemic (1·9% of all screened); 93·0% of RNA-positive individuals initiated DAAs; and 95·8% of those initiated completed treatment. Performance was consistently strong across health facilities, with marked geographic heterogeneity and identifiable hotspots. The principal gap was confirmatory testing (18·2% of anti-HCV-positive individuals lacked RNA testing). We provide policy-ready denominators and equity stratifications (age, sex, facility type, district/province).

**Implications of all the available evidence**

Primary-care delivery within public systems can achieve high initiation and completion and is a feasible path to HCV elimination in resource-limited Western Pacific settings. Closing the confirmatory-testing gap (e.g., reflex RNA or core antigen testing from the same encounter), strengthening sample transport and recall systems, and prioritising low-coverage, high-burden districts—particularly among men and older adults—should accelerate progress. Establishing patient-level linkages to capture sustained virological response at week 12 post-treatment will enable robust cure monitoring during scale-up.

## Introduction

Hepatitis C virus (HCV) remains a major public health problem, with ∼58 million people living with chronic infection and ∼1 million new infections annually.^1^^,^^2^ Chronic HCV infection leads to cirrhosis, hepatocellular carcinoma, and premature mortality. In 2016, the World Health Organisation (WHO) set 2030 elimination targets—90% reduction in new infections and 65% reduction in HCV-related mortality—implying that ∼7·2 million people must be linked to care and started on treatment each year.^1^^,^^2^ Over the past decade, the combination of direct-acting antivirals (DAAs) with streamlined “test-and-treat” pathways has made population-level cure feasible by shortening treatment (typically 8–12 weeks), simplifying pre-treatment assessment, and reducing losses along the care cascade.^3^, ^4^, ^5^ Several countries (e.g., Egypt, Australia, Georgia, France, and Iceland) have reported programmatic progress under such models, while WHO recommends active community case-finding to detect undiagnosed infections and reach higher-risk populations—reinforcing simplified, decentralised delivery within primary care.^1^^,^^6^^,^^7^

In Cambodia, HCV prevalence among the general population was ∼1·2% in 2022, higher than neighbouring countries, including Thailand (0·5%), Laos (0·6%), and Vietnam (0·9%).^8^ In contrast, much of the response in Vietnam and Thailand has focused on micro-elimination programmes targeting high-risk groups.^9^^,^^10^ Liver cancer comprises a large share of new cancer diagnoses in Cambodia.^11^ Previous studies reported 2·6% and 1·9% of anti-HCV and RNA (viraemia) prevalence, respectively, with notably higher prevalence among adults >45 years, consistent with historical iatrogenic exposures (e.g., unsafe medical/dental procedures and transfusions before 1996).^12^^,^^13^ Populations at increased risk include people who inject drugs (PWID) and others with parenteral drug exposures, people living with HIV (PLHIV), individuals with household exposure to someone with HCV, and healthcare workers; among men who have sex with men (MSM), risk is practice-dependent rather than identity-based.^14^^,^^15^ Despite this burden, barriers to access persist—particularly outside urban centres—owing to distance to services, fragmented diagnostic pathways, multiple-visit requirements, and limited confirmatory testing capacity.^12^

In Cambodia, HCV was historically a low priority, with diagnostic and treatment services centralised at specialist centres.^16^ HCV programme began with Médecins Sans Frontières (MSF)–led pilots (2016–2021) of decentralised and simplified models of care^17^^,^^18^; in collaboration with the Ministry of Health (MoH), which subsequently transferred to public services.^19^ Since 2018, the MoH has led a National Viral Hepatitis Technical Working Group and adopted a National Strategic Plan (2020–2024), moving to a government-led scale-up in 2023.^19^ Key responsibilities, including testing, pre-treatment assessment, and DAA initiation, have shifted from specialists to trained healthcare workers, and HCV services have been integrated into public health services in six provinces. The programme has scaled up testing to all adults and prioritises individuals aged ≥45 years and other higher-risk groups through target facility-based testing rather than once-off population screening.^20^

The Viral Hepatitis Elimination Programme is state-funded, with an annual budget of US$1 million, half of which is allocated directly to commodities, including DAAs.^21^^,^^22^ MoH centralised procurement covers HCV rapid diagnostic tests (RDTs), laboratory consumables and DAAs, with market-shaping/procurement and technical support from Clinton Health Access Initiative (CHAI) and WHO to leverage global access prices.^23^ Global Fund has supported HCV screening and treatment for PLHIV co-infected with HCV.^24^ Looking ahead, sustainability rests on further integration into primary health care per WHO's simplified service delivery guidance,^3^ progressive increases in domestic financing, and maintaining affordability through multi-year price/volume agreements; WHO, CHAI, and development partners (including Expertise France/L'Initiative) are providing ongoing technical assistance for guidelines updates, planning, and costing.^21^^,^^22^

Although MSF pilots demonstrated high virological cure under a simplified approach,^16^ the performance of Cambodia's national decentralised model under routine conditions has not been assessed using standardised cascade indicators across multiple provinces and facility types. Key uncertainties include confirmatory testing uptake, linkage to treatment among RNA-positive patients, treatment completion, and equity across age, sex, facility type, and geography. An early programme-level evaluation is therefore warranted to quantify cascade performance, identify bottlenecks, and guide optimisation during scale-up.

We aimed to assess the early performance of Cambodia's decentralised, primary-care HCV strategy across six provinces in 2024 by quantifying the cascade from screening to confirmatory testing, treatment initiation, and treatment completion. Secondary objectives were to characterise attrition along the pathway and explore equity across age, sex, facility type, operational district, and province.

## Methods

### Study design

We conducted a retrospective observational analysis of routinely collected data from Cambodia's National Viral Hepatitis Elimination Programme, coordinated by the Communicable Disease Control Department (CDC) of the MoH. Reporting followed the STROBE guidelines with pre-specified cascade indicators, explicit denominators, and a priori stratification for equity analyses.

### Setting

Cambodia's health system comprises 103 operational health districts within 25 municipal/provincial health departments, served by referral hospitals and health centres.^25^ In 2024, the National Viral Hepatitis Elimination Programme operated in 15 operational districts across six provinces, with all public health facilities in these districts participating: 238 health centres and 18 referral/provincial hospitals.^19^ Testing and treatment sites were selected pragmatically based on the HCV burden, and health-system readiness, rather than random sampling.^16^^,^^19^^,^^26^ The national roll-out launched in November 2023, with activities introduced progressively as districts and health facilities became operationally ready, achieving full implementation by January 2024.

### Participants

The study included all persons accessing HCV services at participating facilities between 1 January and 31 December 2024. Consistent with national guidance, testing and treatment were offered to all adults, prioritising those aged ≥45 years, healthcare workers, PLHIV, MSM, PWID or with other parenteral drug exposures, and individuals with household exposure to a family member with HCV.^20^ Testing procedures were standardised across health facilities, thereby allowing patients to choose testing facilities within their districts. Analyses of coverage and cascade proportions were restricted to adults (≥18 years).

### Programme model and care pathway

HCV testing, pre-treatment assessment, and DAA initiation were performed at the point-of-care by trained clinical staff.^20^ Before the national roll-out, the CDC collaborated with the WHO to provide a series of standardised trainings covering testing strategies and algorithms, treatment protocols, and data reporting procedures (Supplementary Material; Figure S1).^20^ Testing was voluntary, with staff providing information about viral hepatitis and obtaining verbal consent. Individuals had the right to decline testing. Health facilities implemented a user-fee structure for HCV services: 5000 riels (US$1·25) for HCV RDT/consultation, 20,000–78,000 riels (US$5–19·5) for HCV RNA confirmatory testing, and 10,000–20,000 riels (US$2·5–5) for four weeks of treatment and each refill. Fees were waived for holders of the government-issued identification poor and/or health equity cards.

**Fig. 1:**
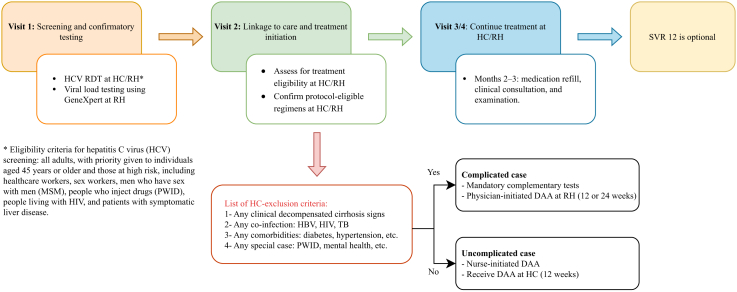
Stages of the hepatitis C (HCV) care cascade and patient flow in the Cambodian National Viral Hepatitis Elimination Programme. Abbreviations: RDT, rapid diagnostic test; DAA, direct-acting antiviral (Sofosbuvir+Daclatasvir); HC, health centre; RH, referral hospital; HIV, human immunodeficiency virus; HBV, hepatitis B virus; M, month; TB, tuberculosis.

The standardised pathway comprised: (i) point-of-care anti-HCV RDT WHO-prequalified assays (SD Bioline HCV, Abbott Diagnostics, Korea); (ii) automatic eligibility of first-time anti-HCV-reactive individuals for confirmatory HCV RNA testing (PCR) at designated laboratories using GeneXpert® HCV viral load test (Cepheid, Sunnyvale, USA); (iii) simplified pre-treatment assessment without genotyping (use of non-invasive fibrosis scores); and (iv) initiation of pan-genotypic direct-acting antivirals at health centres for uncomplicated cases, with referral hospitals managing complicated cases by physicians (e.g., decompensated cirrhosis, HIV/TB co-infection, major comorbidities; Fig. 1). Where feasible, same-day or rapid initiation was encouraged. In routine practice, the pathway typically required four visits, from screening to treatment completion. Given the programme start and the 2024 observation window, sustained virological response post-treatment at week 12 (SVR12) was not assessed systematically; primary outcomes therefore stop at treatment initiation and completion.

### Data sources and management

All data were extracted from CDC. Facilities submitted monthly, facility-level aggregate reports (Excel) to the national programme. For each facility-month and by sex and age group, the dataset included counts of: individuals screened, anti-HCV-reactive, underwent RNA testing, RNA detectable, initiated DAA, completed treatment, and lost to follow-up (LTFU). Data on patients tested negative for anti-HCV were not recorded or reported to the national programme. Data were anonymised at source and contained no direct identifiers. We applied routine data quality procedures (indicator dictionary, range and internal-consistency checks, reconciliation with laboratory tallies) and excluded facility-months failing predefined completeness thresholds; details are provided in the supplement.

The population denominators were obtained from operation district data based on the Cambodia Population Projection 2020–2023 (National Institute of Statistics, Ministry of Planning), derived from the General Population Census 2019, and the Cambodia Health Demographic Survey 2021–2022.^27^

### Outcomes and definitions

We evaluated the HCV cascade of care using explicit denominators:

1. Testing coverage: number of individuals tested among the operational district population ≥18 years (estimated from official projections); coverage is reported in sensitivity analyses.
2. Anti-HCV positivity: anti-HCV-reactive among those tested.
3. RNA testing: proportion of anti-HCV-reactive individuals with HCV RNA PCR results.
4. Viraemia: RNA-detectable among those with RNA testing (assay and reporting thresholds per programme laboratory protocols). HCV RNA-detectable was classified if the viral load was within quantifiable range (10–100,000,000 IU/mL), or if HCV RNA was present but below the lower limit of quantification (<10 IU/mL). Only results reported by GeneXpert® as “HCV NOT DETECTED” were classified as negative. If a test result was invalid, the blood sample was retested, or patient was contacted to provide a new specimen.
5. Treatment initiation: proportion of RNA-positive individuals who started DAAs.
6. Treatment completion: completion of a 12- or 24-week DAA regimen within protocol-defined windows among those initiated. Following the dispensing of the full DAA course, completion was confirmed via telephone by healthcare workers.
7. LTFU: no attendance ≥90 days beyond a scheduled confirmatory testing or treatment visit, per programme rules.

### Explanatory variables

Pre-specified covariates were age (≤45 versus >45 years), sex (female versus male), facility type (health centre versus referral hospital), operational district and province, and calendar quarter (January–March [Q1], April–June [Q2], July–September [Q3], and October–December [Q4], 2024).

### Statistical analysis

All analyses acknowledged the hierarchical structure of the data. We treated the operational district as the highest clustering level (primary sampling unit), with facilities nested within operational districts for multi-stage analyses. Descriptive proportions (tested among eligible, RNA tested among anti-HCV–positive, DAA initiation, and completion) were estimated using design-based survey estimators with logit-transformed 95% confidence intervals (CIs). Group comparisons used Rao–Scott adjusted χ^2^ (F) or design-based Wald tests. The relationship between anti-HCV positivity and viraemia at operational district level was explored using Spearman's rank correlation.

For regression, adjusted odds ratios (aORs) were estimated using design-based generalised linear models (GLMs), which accounted for the multi-stage survey design. The rollout was a near-census of sites; consequently, sampling weights were set to one (self-weighted design). Degrees of freedom followed survey design conventions (df = number of ODs − number of strata), and single-cluster strata were handled using the standard lonely primary sampling unit (PSU) adjustment. In panel analyses, we included province fixed effects and calendar quarter indicators to capture unobserved heterogeneity and time trends.

Pre-specified sensitivity analyses included: (i) restriction to facilities with ≥9 months of reporting; (ii) exclusion of Q1 as run-in; (iii) alternative LTFU windows (60/120 days); and (iv) alternative coverage denominators (adults ≥18 years). A two-sided p < 0·050 threshold denoted statistical significance. Analyses were conducted in R (v4·4·1),^28^ and maps were produced in QGIS 3·38 using national shapefiles.^29^

### Ethics approval

This study analysed de-identified programme aggregates under data-sharing agreements with the MoH and received ethics approval from the National Ethics Committee for Health Research, Cambodia (approval number 036 NECHR; 28 November 2024). Individual consent was waived in accordance with regulations on secondary analysis of routine data.

### Role of funders

The programme is government-funded; external funders had no role in study design, data collection, analysis, interpretation, or writing.

## Results

### Testing and population coverage

**Fig. 2:**
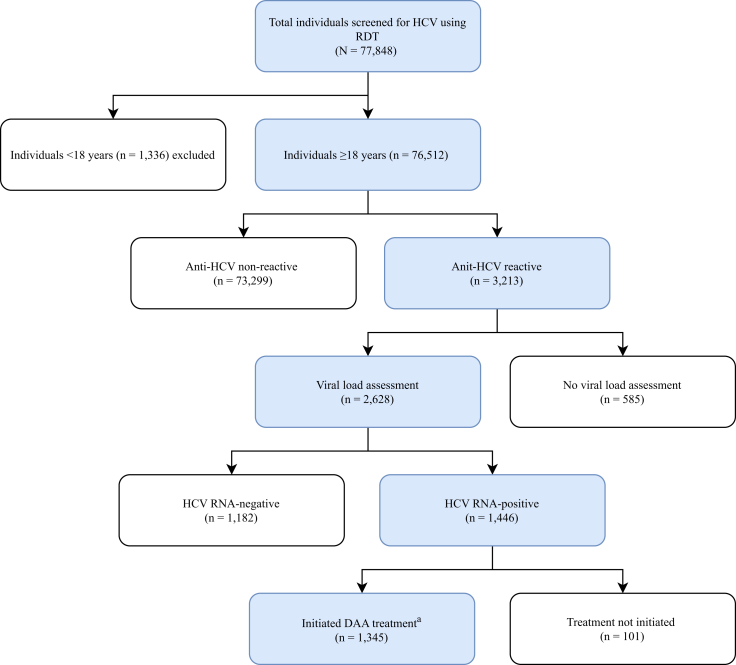
Study flow diagram of participants. ^a^DAA, direct-acting antiviral (Sofosbuvir + Daclatasvir).

**Fig. 3:**
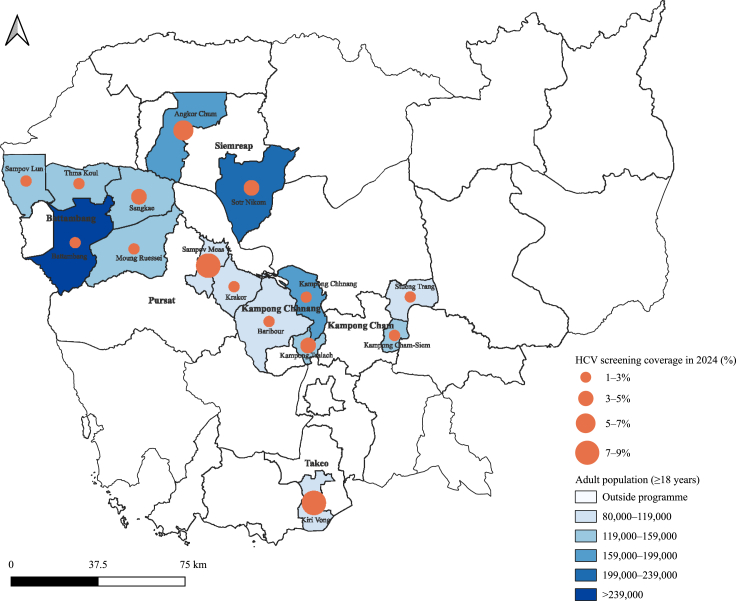
Geographic distribution of adult population (≥18 years) and hepatitis C virus (HCV) testing uptake by operational district in 2024.

From January to December 2024, 77,848 individuals were tested for anti-HCV across 15 operational districts; 76,512 (98·2%) were adults (≥18 years) and formed the analytical population (Fig. 2). The median number of tests per operational districts was 4074 (IQR 2564–8541), and 71·6% (54,757/76,512) of tests were performed at health centres. Using adult operational district denominators (2,196,351), overall testing coverage was 3·5% (95% CI 2·5–4·4), ranging from 1·8% in Thma Koul to 8·7% in Sampov Meas (Table 1; Fig. 3).

**Table 1:** The hepatitis C testing coverage in 15 operational districts in six provinces across Cambodia from January to December 2024.

Province	Operational district (OD)	Health facilities		Adults aged ≥18 years	HCV testing coverage		Anti-HCV positive	
		HCs	RHs		n/N (%)	95% CI	n/N (%)	95% CI
Overall		n = 238	n = 18	N = 2,196,351	76,512/2,196,351 (3·5)	2·5–4·4	3213/76,512 (4·2)	3·1–5·6
Kampong Cham	Kampong Cham-Siem	16	1	125,689	3309/125,689 (2·6)	1·5–4·5	58/3309 (1·8)	0·3–9·0
	Stueng Trang	12	1	95,994	2155/95,994 (2·2)	1·6–3·1	48/2155 (2·2)	1·0–5·0
Kampong Chhnang	Kampong Chhnang	20	1	188,839	4074/188,839 (2·2)	1·6–2·9	359/4074 (8·8)	3·7–19·4
	Kampong Tralach	15	1	148,563	4545/148,563 (3·1)	2·2–4·3	66/4545 (1·5)	0·6–3·3
	Baribour	10	1	89,756	2365/89,756 (2·6)	1·5–4·7	329/2365 (13·9)	7·1–25·6
Battambang	Sampov Lun	10	1	127,094	2564/127,094 (2·0)	1·0–4·0	195/2564 (7·6)	2·4–21·4
	Thma Koul	17	2	156,279	2856/156,279 (1·8)	1·2–2·7	206/2856 (7·2)	3·6–13·8
	Battambang	28	1	263,031	7865/263,031 (3·0)	1·9–4·6	388/7865 (4·9)	2·2–10·9
	Sangkae	14	2	132,977	4585/132,977 (3·4)	2·8–4·3	142/4585 (3·1)	1·4–6·7
	Moung Ruessei	14	1	154,944	3074/154,944 (2·0)	1·2–3·2	80/3074 (2·6)	1·2–5·5
Pursat	Krakor	11	1	83,826	2515/83,826 (3·0)	1·7–5·2	210/2515 (8·3)	2·3–26·2
	Sampov Meas	12	1	100,250	8734/100,250 (8·7)	3·1–22·1	207/8734 (2·4)	0·7–7·8
Siem Reap	Angkor Chum	21	2	196,879	10,603/196,879 (5·4)	3·4–8·5	426/10,603 (4·0)	1·5–10·5
	Sotr Nikum	25	1	225,382	8727/225,382 (3·9)	1·9–7·9	377/8727 (4·3)	1·7–10·7
Takeo	Kiri Vong	13	1	106,846	8541/106,846 (8·0)	4·3–14·3	122/8541 (1·4)	0·5–3·7

Abbreviations: HC, health centre; RH, referral hospital; CI, confidence interval. Data are presented as n/N (%).

Denominators—Testing coverage: N = adult population (≥18 years); Anti-HCV positivity: N = tested individuals. Analyses accounted for the multi-stage design, with facilities nested within operational districts. 95% CIs were calculated using design-based survey estimators with logit-transformed.

### Antibody positivity and confirmatory testing

Overall, 3213/76,512 (4·2%; 95% CI 3·1–5·6) individuals were anti-HCV–positive (median per operational district: 206; IQR 80–359), with substantial heterogeneity across operational districts (p < 0·0001): 1·4% in Kiri Vong versus 13·9% in Baribour. Anti-HCV positivity was more frequent among women (1963/3213, 61·1%) and adults >45 years (2407/3213, 74·9%), and over half were identified at health centres (1836/3213, 57·1%). Among anti-HCV–positive individuals, 2628/3213 (81·8%, 95% CI 74·4–87·4) underwent confirmatory HCV RNA testing (Table 2). The proportion of RNA testing was highest in quarter 2 (Q2: 732/756, 97·5%) and Q1 (792/911, 86·9%; p < 0·0001), and in Battambang province (891/1011, 88·1%), Kampong Cham (91/106, 85·5%), and Kampong Chhnang (358/425, 84·2·4%; p < 0·0001). In total, 585/3213 (18·2%) anti-HCV–positive individuals did not undergo confirmatory testing; most were female (351/585, 60·0%), >45 years (365/585, 62·4%), and had been tested at health centres (308/585, 52·7%).

**Table 2:** Hepatitis C virus (HCV) cascade outcomes among anti-HCV positive individuals, by subgroups across 15 operational districts, Cambodia, 2024.

Variables	Anti-HCV positive n/N (%)^a^	Viral load assessed n/N (%)^b^	p-value	RNA-positive n/N (%)^c^	p-value	Treatment initiation n/N (%)^d^	p-value	Treatment completion n/N (%)^e^	p-value
Overall	3213	2628		1446		1345		1289	
**Age group**			0·013		0·146		0·222		0·477
18–45 Y	806/3213 (25·1)	586/806 (72·7)		301/586 (51·4)		298/301 (99·0)		288/298 (96·6)	
>45 Y	2407/3213 (74·9)	2042/2407 (84·8)		1145/2042 (56·1)		1047/1145 (91·4)		1001/1047 (95·6)	
**Sex**			0·917		0·003		0·951		0·062
Female	1963/3213 (61·1)	1612/1963 (82·1)		835/1612 (51·8)		787/835 (94·3)		761/787 (96·7)	
Male	1250/3213 (38·9)	1016/1250 (81·3)		611/1016 (60·1)		558/611 (91·3)		528/558 (94·6)	
**Health facility**			0·460		0·193		0·541		0·004
Health centre	1836/3213 (57·1)	1528/1836 (83·2)		871/1528 (57·0)		813/871 (93·3)		802/813 (98·6)	
Referral hospital	1377/3213 (42·9)	1100/1377 (79·9)		575/1100 (52·3)		532/575 (92·5)		487/532 (91·5)	
**Calendar quarter**			<0·0001		0·003		0·911		0·004
Q1 (Jan–Mar)	911/3213 (28·4)	792/911 (86·9)		395/792 (49·9)		361/395 (91·4)		353/361 (97·8)	
Q2 (Apr–Jun)	756/3213 (23·5)	737/756 (97·5)		433/737 (58·8)		406/433 (93·8)		398/406 (98·0)	
Q3 (Jul–Sep)	891/3213 (27·7)	596/891 (66·9)		314/596 (52·7)		296/314 (94·3)		275/296 (92·9)	
Q4 (Oct–Dec)	655/3213 (20·4)	503/655 (76·8)		304/503 (60·4)		282/304 (92·8)		263/282 (93·3)	
**Province**			<0·0001		<0·0001		<0·0001		<0·0001
Battambang	1011/3213 (31·5)	891/1011 (88·1)		470/891 (52·7)		401/470 (85·3)		366/401 (91·3)	
Kampong Cham	106/3213 (3·3)	91/106 (85·8)		63/91 (69·2)		58/63 (92·1)		58/58 (100·0)	
Kampong Chhnang	425/3213 (13·2)	358/425 (84·2)		174/358 (48·6)		173/174 (99·4)		169/173 (97·7)	
Pursat	746/3213 (23·2)	587/746 (78·7)		292/587 (49·7)		283/292 (96·9)		275/283 (97·2)	
Siem Reap	803/3213 (25·0)	621/803 (77·3)		387/621 (62·3)		374/387 (96·6)		366/374 (97·9)	
Takeo	122/3213 (3·8)	80/122 (65·6)		60/80 (75·0)		56/60 (93·3)		55/56 (98·2)	
**Operational district**			<0·0001		<0·0001		<0·0001		<0·0001
Kampong Cham-Siem	58/3213 (1·8)	56/58 (96·6)		40/56 (71·4)		37/40 (92·5)		37/37 (100·0)	
Stueng Trang	48/3213 (1·5)	35/48 (72·9)		23/35 (65·7)		21/23 (91·3)		21/21 (100·0)	
Kampong Chhnang	359/3213 (11·2)	300/359 (83·6)		141/300 (47·0)		140/141 (99·3)		136/140 (97·1)	
Kampong Tralach	66/3213 (2·1)	58/66 (87·9)		33/58 (56·9)		33/33 (100·0)		33/33 (100·0)	
Baribour	329/3213 (10·2)	272/329 (82·7)		119/272 (43·8)		117/119 (98·3)		113/117 (96·6)	
Sampov Lun	195/3213 (6·1)	165/195 (84·6)		71/165 (43·0)		55/71 (77·5)		53/55 (96·4)	
Thma Koul	206/3213 (6·4)	159/206 (77·2)		92/159 (57·9)		89/92 (96·7)		83/89 (93·3)	
Battambang	388/3213 (12·1)	370/388 (95·4)		218/370 (58·9)		172/218 (78·9)		147/172 (85·5)	
Sangkae	142/3213 (4·4)	132/142 (93·0)		61/132 (46·2)		58/61 (95·1)		56/58 (96·6)	
Moung Ruessei	80/3213 (2·5)	65/80 (81·3)		28/65 (43·1)		27/28 (96·4)		27/27 (100·0)	
Krakor	210/3213 (6·5)	197/210 (93·8)		91/197 (46·2)		88/91 (96·7)		86/88 (97·7)	
Sampov Meas	207/3213 (6·4)	118/207 (57·0)		82/118 (69·5)		78/82 (95·1)		76/78 (97·4)	
Angkor Chum	426/3213 (13·3)	369/426 (86·6)		243/369 (65·9)		235/243 (96·7)		229/235 (97·4)	
Sotr Nikum	377/3213 (11·7)	252/377 (66·8)		144/252 (57·1)		139/144 (96·5)		137/139 (98·6)	
Kiri Vong	122/3213 (3·8)	80/122 (65·6)		60/80 (75·0)		56/60 (93·3)		55/56 (98·2)	

Abbreviations: HCV, hepatitis C virus; RNA, ribonucleic acid. Data are presented as n/N (%). The proportion was calculated based on the denominator preceding the relevant HCV cascade step.

Group comparisons used design-based Wald tests accounting for clustering.

P-value were two-sided and derived from design-based Wald F tests using design-based generalised linear models, accounting for the multi-stage design with health facilities nested within operational districts.

a Anti-HCV positive: number of individuals who tested positive for anti-HCV (n) divided by the total number of individuals tested (N).

b Viral load assessed: number of individuals who underwent confirmatory viral load assessment (n) divided by the number of individuals who tested positive for anti-HCV (N) in that subgroup.

c RNA-positive: number of individuals with detectable HCV RNA divided by the number of individuals who underwent viral load confirmatory assessment (N) in that subgroup.

d Treatment initiation: number of individuals with RNA-positive who initiated treatment (n) divided by number of RNA-positive (N) in that subgroup.

e Treatment completion: number of individuals completed full course of treatment (n) divided by the number of individuals who initiated treatment (N) in that subgroup.

### Viraemia

Among those with RNA results, 1446/2628 (55·0%; 95% CI 49·5–60·3) were RNA-positive (median per operational district: 82; IQR 40–141). Viraemia proportions were higher among men (611/1016 versus 835/1612; 60·1% versus 51·8%, p = 0·003), and varied over time (Q4 304/503, 60·4%, p = 0·003). Provincial proportions were highest in Takeo (60/80, 75·0%), Kampong Cham (63/91, 69·2%), and Siem Reap (387/621, 62·3%) (Table 2). Across operational districts, anti-HCV positivity and viraemia were inversely correlated (Spearman ρ = −0·618; p = 0·016), an ecological finding consistent with a higher share of resolved infections where antibody prevalence is high.

### Linkage to care and treatment initiation

Of RNA-positive individuals, 1345/1446 (93·0%; 95% CI 85·8–96·7) initiated DAAs therapy. Uptake exceeded 95% in Kampong Chhnang province (173/174, 99·4%), Pursat (283/292, 96·9%), and Siem Reap (374/387, 96·6%; p < 0·0001). The treatment uptake was high among women (787/835, 94·3% versus 558/611, 91·3%; p = 0·951) and in adults 18–45 years (298/301, 99·0% versus 1047/1145, 91·4%; p = 0·222). Uptake was similar by facility level (813/871; 93·3% at health centres versus 532/575; 92·5% at referral hospitals; p = 0·541).

### Treatment completion

Among those initiating treatment, 1289/1345 (95·8%; 95% CI 91·7–97·9) completed the DAA course. Completion was greater at health centres (802/813, 98·6%) than at referral hospitals (487/532, 91·5%; p = 0·004) and varied by province (Kampong Cham 58/58, 100% versus Battambang 366/401, 91·3%; p < 0·0001). Reasons for non-completion were not documented.

### Factors associated with viraemia, treatment initiation, and completion

In multivariable, design-based GLMs using facility-month aggregates (Table 3), men had higher odds of viraemia (aOR 1·40; 95% CI 1·05–1·86). Compared with Q1, odds of viraemia were greater in Q4 (1·79; 95% CI 1·05–3·06), and varied by province (Takeo aOR 2·79; 95% CI 1·87–4·18; Kampong Cham aOR 2·11; 95% CI 1·27–3·51).

**Table 3:** Univariate and multivariate associations of selected subgroups with viraemia, treatment initiation, and completion.

Variables	Viraemia (n = 2628)				Treatment initiation (n = 1446)				Treatment completion (n = 1289)			
	OR (95% CI)	p-value	aOR (95% CI)	p-value	OR (95% CI)	p-value	aOR (95% CI)	p-value	OR (95% CI)	p-value	aOR (95% CI)	p-value
**Age group**												
18–45 Y	Ref.	0·146	Ref.	0·250	Ref.	0·222	Ref.	0·751	Ref.	0·477	Ref.	0·619
>45 Y	1·21 (0·93–1·56)		1·15 (0·84–1·58)		0·76 (0·48–1·21)		0·93 (0·49–1·78)		0·75 (0·32–1·75)		0·78 (0·19–3·20)	
**Sex**												
Female	Ref.	0·003	Ref.	0·032	Ref.	0·951	Ref.	0·509	Ref.	0·062	Ref.	0·375
Male	1·40 (1·14–1·72)		1·40 (1·05–1·86)		1·01 (0·78–1·29)		1·08 (0·78–1·51)		0·60 (0·35–1·03)		0·72 (0·27–1·96)	
**Health facility**												
Referral hospital	Ref.	0·193	Ref.	0·082	Ref.	0·541	Ref.	0·732	Ref.	0·004	Ref.	0·052
Health centre	1·20 (0·90–1·61)		1·37 (0·93–2·04)		1·32 (0·51–3·43)		1·17 (0·31–4·33)		6·72 (2·05–22·01)		6·05 (0·98–37·53)	
**Calendar quarter**												
Q1 (Jan–Mar)	Ref.	0·003	Ref.	0·070	Ref.	0·911	Ref.	0·909	Ref.	0·004	Ref.	0·181
Q2 (Apr–Jun)	1·40 (1·05–1·86)		1·47 (0·99–2·20)		1·00 (0·46–2·21)		0·88 (0·28–2·76)		1·12 (0·11–11·42)		0·96 (0·03–31·79)	
Q3 (Jul–Sep)	1·12 (0·79–1·58)		1·25 (0·77–2·03)		1·08 (0·42–2·78)		1·04 (0·30–3·56)		0·30 (0·09–0·98)		0·33 (0·06–1·90)	
Q4 (Oct–Dec)	1·53 (1·09–2·16)		1·79 (1·05–3·06)		0·79 (0·25–2·53)		0·77 (0·21–2·85)		0·32 (0·05–1·82)		0·41 (0·03–4·98)	
**Province**												
Battambang	Ref.	<0·0001	Ref.	0·005	Ref.	<0·0001	Ref.	0·016	–	–	–	–
Kampong Cham	1·97 (1·40–2·79)		2·11 (1·27–3·51)		3·09 (2·38–4·00)		3·11 (1·79–5·39)		–	–	–	–
Kampong Chhnang	0·85 (0·60–1·20)		0·80 (0·52–1·23)		2·05 (1·43–2·92)		2·08 (1·20–3·60)		–	–	–	–
Pursat	0·90 (0·51–1·57)		0·86 (0·36–2·04)		3·76 (2·46–5·75)		3·69 (2·00–6·82)		–	–	–	–
Siem Reap	1·50 (0·98–2·28)		1·46 (0·85–2·49)		2·13 (1·53–2·97)		2·09 (1·44–3·04)		–	–	–	–
Takeo	2·73 (2·01–3·70)		2·79 (1·87–4·18)		0·99 (0·78–1·26)		0·96 (0·73–1·27)		–	–	–	–

Abbreviations: OR, odds ratio; aOR, adjusted odds ratio; CI, confidence interval; Q, calendar quarter.

All models used design-based logistic regression to account for multi-stage sampling design, with clustering at operational districts and nesting at health facility levels.

In treatment completion model, province was included as a fixed effect to control for confounding; however, its OR/aOR was not reported due to rare event analysis (lost to follow-up during treatment).

For multi-category variables (calendar quarter and province), the global p-value was reported from a joint design-based Wald F test.

For treatment initiation, aORs were elevated in Pursat (aOR 3·69; 95% CI 2·00–6·82), Kampong Cham (aOR 3·11; 95% CI 1·79–5·39), Siem Reap (aOR 2·09; 95% CI 1·44–3·04), and Kampong Chhnang (aOR 2·08; 1·20–3·60); no independent associations were observed for health facility type, age, sex, and calendar quarter after adjustment. Treatment completion was higher at health centres (aOR 6·05; 95% CI 0·98–37·53; p = 0·082), but this was not statistically significant.

### Cascade yield (summary)

**Fig. 4:**
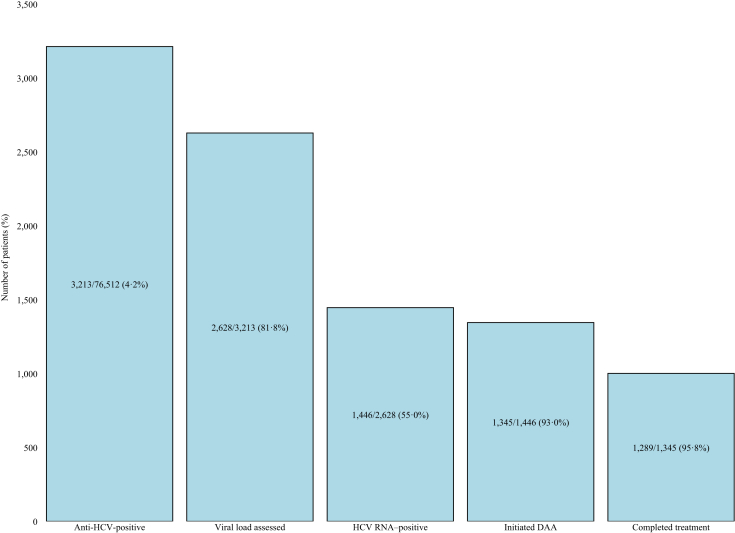
Hepatitis C virus (HCV) cascade of care (Tested → anti-HCV–positive → RNA assessment → RNA positive → initiated DAA → completed treatment) between January and December 2024.

Among adults tested (N = 76,512), 3213 (4·2%) were anti-HCV–positive. Of the 2628 with RNA testing, 1446 (55·0%) were RNA-positive (1·9% of all screened). Among RNA–positive individuals, 1345/1446 (93·0%) initiated DAA and 1289/1345 (95·8%) completed treatment (89·1% of RNA–positive; Fig. 4). Expressed per 100 adults screened: ∼4·2 anti-HCV–positive, 1·9 RNA positive, 1·8 initiated treatment, and 1·7 completed treatment.

## Discussion

In this early multi-province implementation of Cambodia's decentralised, primary-care HCV programme, we observed a well-functioning cascade with strong downstream performance under routine conditions. Among 76,512 adults tested, 4·2% were anti-HCV–positive; 81·8% of seropositive individuals received confirmatory RNA testing; 55·0% of those tested were viraemic (1·9% of all screened); 93·0% of RNA-positive individuals initiated DAA; and 95·8% of those initiated completed treatment. HCV testing coverage reached 3·5% of the adult population across operational districts, with marked geographical heterogeneity. Viraemia was more frequent among men and varied by province and calendar quarter. Under the decentralised approach, Pursat, Kampong Cham, Siem Reap, and Kampong Chhnang provinces reported consistently high rates of treatment initiation.

These findings support the feasibility of simplified primary-care delivery as a cornerstone of HCV elimination, aligning with WHO's 2030 targets^1^^,^^2^ and evidence that streamlined test-and-treat pathways coupled with short-course, pan-genotypic DAAs can reduce attrition and enable cure at scale compared with a centralised model (Supplementary Material, Table S1).^3^^,^^6^^,^^18^^,^^30^^,^^31^ Countries that have scaled such models—Egypt, Australia, Georgia, France, and Iceland—have documented progress towards elimination, providing external validity for our observations.^6^^,^^7^^,^^32^^,^^33^ The main attrition point in our cascade was confirmatory testing (18·2% of anti-HCV–positive individuals lacked RNA testing). These patterns were not investigated with inferential statistics as demographic data were not available for individuals with anti-HCV–negative. Preventing drop-off at the confirmatory step which is expected in multi-visit pathways and directly actionable: reflex confirmatory testing (RNA or core antigen) at the same encounter, stronger specimen transport systems, and recall for pending results are likely to yield immediate gains.^1^^,^^3^^,^^34^

Marked geographical heterogeneity in anti-HCV prevalence was observed, with the highest proportions in Battambang, Pursat, and Kampong Chhnang. These provinces have previously been identified as hotspots of HCV infection, may reflect historic transmission during/post-war (1975–1991), iatrogenic contamination, and traditional incision practices.^12^^,^^13^^,^^35^ In addition, variation in HCV burden and programme performance is likely influenced by patient-and health facility-level factors, such as patient education, staffing, geographic barriers, and health system constraints.^36^^,^^37^ Consistent with a recent decentralised study, age, sex, and facility type were associated with diagnosis, viraemia, treatment initiation, and completion.^38^ The inverse ecological correlation between anti-HCV positivity and viraemia across districts is compatible with older infection cohorts and a higher share of resolved or previously treated infections in areas with historically greater exposure, although age structure and prior testing/treatment patterns may also contribute; causal inferences should be avoided.

The treatment initiation and completion rates surpassed 90%, with comparable performance across health facilities. This reflects the benefits of proximity, streamlined pathways, and fewer visits, suggesting a role for patient navigation, differentiated follow-up, and rapid down-referral after stabilisation.^1^^,^^39^ Our findings extend programme-embedded evidence generated by MSF pilots in Cambodia, where simplified regimens achieved high virological cure,^16^^,^^17^ to a national decentralised model operating across multiple provinces and facility types. Unlike the pilots, our 2024 window does not include SVR12 ascertainment; nonetheless, the near-universal initiation among RNA-positive individuals and very high completion among those initiated are consistent with conditions necessary to achieve high cure rates once systematic SVR12 follow-up is implemented. This is supported by extensive real-world data demonstrated that DAAs therapy achieved >95% SVR12,^16^ including in HIV/HCV co-infection.^40^

Strengths include real-world, multi-province coverage; standardised cascade indicators with explicit denominators; and policy-relevant stratifications by sex, age, facility type, and district/province. Limitations arise from the use of aggregate facility-month data: demographics of anti-HCV–negative cases, and data on high-risk behaviours were not routinely captured by the programme. Testing coverage was estimated using the population aged ≥18 years, which likely underestimates the national programme performance in priority target groups (≥45 years). Although PWID in Cambodia exhibited a high prevalence of anti-HCV (30·4%) and HIV/HCV co-infection (9·4%), this population is relatively small compared with neighbouring countries; therefore, our findings may not generalise to settings where injecting drug use is widespread.^41^^,^^42^ Individuals could not be de-duplicated across sites; however, repeat testing was minimal because population testing was limited to one test per person. Reasons for non-initiation or non-completion were not captured, and individual time-to-event metrics could not be estimated. Site selection was pragmatic (15 operational districts) and may limit generalisability to non-participating areas. Multiple comparisons may increase false-positive findings; we therefore emphasised effect sizes with 95% CIs using design-based survey methods and pre-specified contrasts. Limited individual-level data prevented further analysis of risk factors and district heterogeneity. Finally, although final models included province fixed effects, province-specific predictors of treatment completion were not reported because discontinuation events were rare in several provinces.

### Implications for policy and practice

Three near-term actions emerge. *(i) Close the confirmatory gap*: implement reflex confirmatory testing from the same encounter where feasible, and strengthen specimen transport and result-return systems. *(ii) Consolidate primary-care delivery*: preserve initiation and follow-up at health centres while supporting referral hospitals with case management for complicated patients and rapid down-referral. *(iii) Plan scale-up and equity*: coverage of 3·5% indicates early scale—expansion should prioritise districts with low coverage and high viraemia proportions, and maintain focus on men and older adults where viraemia risk is higher.^43^

### Priorities for measurement and research

For 2025+, the programme should: (1) enable patient-level linkage between clinic and laboratory systems to capture SVR12 systematically; (2) define eligibility denominators for cure (all who completed treatment with the SVR12 window elapsed); (3) monitor time intervals between cascade steps using routinely captured timestamps; (4) conduct qualitative audits of non-initiation and non-completion; and (5) assess cost and budget impact of reflex testing and further decentralisation. Where laboratory capacity is constrained, evaluation of point-of-care confirmatory options and dried-blood-spot pathways is warranted.^1^^,^^3^

In conclusion, in its first year, Cambodia's decentralised HCV programme achieved high treatment initiation and completion among RNA-positive patients, with confirmatory testing now the principal opportunity for improvement. Strengthening reflex confirmation and consolidating primary-care delivery should accelerate progress as the programme scales. Establishing patient-level linkage to ascertain SVR12 is the critical next step to document cure at population level and benchmark progress towards elimination.

## Contributors

CS and EM contributed to conceptualisation. CS, FG, and EM contributed to study design. SK and SL led programme implementation and provided resources for the National Viral Hepatitis Elimination Programme. KK provided clinical expertise and contributed to interpretation. LST provided methodological input and critical review. CP provided critical review. CS curated the data, conducted data checking, and performed the statistical analyses; FG co-led and supervised the analyses. CS drafted the manuscript. CS and FG had access to raw data and verified the underlying data. All authors contributed to manuscript review and editing. EM had the final responsibility for the decision to submit for publication.

## Data sharing statement

The data was not publicly accessible because of ethical considerations. However, these data will be made available upon reasonable request. Researchers interested in accessing data should submit a formal written request to the Communicable Disease Control Department, Ministry of Health, Cambodia.

## Editor note

The Lancet Group takes a neutral position with respect to territorial claims in published maps and institutional affiliations.

## Declaration of interests

The authors declare no competing interests.
