# Supplementary material for: Early performance of a decentralised, primary-care hepatitis C programme in Cambodia: a retrospective programme evaluation, 2024
Source: Lancet Reg Health West Pac. 2025 Nov 26;65:101758. doi: 10.1016/j.lanwpc.2025.101758 (PMC12689191; doi:10.1016/j.lanwpc.2025.101758)
Supplement: Supplementary Materials [file mmc1.docx]

**​​Supplementary Material**

**Early performance of a decentralised, primary-care hepatitis C programme in Cambodia: a retrospective programme evaluation, 2024**

**Authors:** Chansovannara Soputhy^1,2*^, Florian Girond^2^, Samley Keo^2^, Kolveasna Kim^3,4,5^, Luis Sagaon-Teyssier^1,4^, Capucine Penicaud^5^, Sovann Ly^2^, Emilie Mosnier^1,6^

**Authors’ Affiliations:**

^1^Aix Marseille Univ, Inserm, IRD, SESSTIM, Sciences Economiques & Sociales de la Santé & Traitement de l'Information Médicale, ISSPAM, Marseille, France

^2^ Department of Communicable Disease Control, Ministry of Health, Phnom Penh, Cambodia

^3^ Calmette Hospital, Phnom Penh, Cambodia

^4^ University of Health Sciences, Phnom Penh, Cambodia

^5^ Health Management Support Team (HMST), France

^6^ Infectious and Tropical Diseases Unit, University Hospital of La Réunion, La Réunion, France

Supplementary Table of Contents

[National response to hepatitis C virus (HCV) in Cambodia 1](#_Toc211262899)

[Data Source 3](#_Toc211262900)

[Data Storage and Security 3](#_Toc211262901)

[Data Quality Procedures 3](#_Toc211262902)

[Data Exclusion Criteria 4](#_Toc211262903)

[Data Cleaning Procedure 4](#_Toc211262904)

[Documentation 4](#_Toc211262905)

[References 6](#_Toc211262906)

Supplementary Tables

[**Table S1:** Comparison of pre-2024 centralised versus 2024 decentralised model of hepatitis C (HCV) and HIV/HCV co-infection programme. 2](#_Toc211263359)

[**Table S2:** Proxy subgroup trend among people living with HIV (PLHIV) in HIV clinics in 2024 2](#_Toc211263360)

Supplementary Figures

[**Figure S1:** Hepatitis C virus (HCV) testing and treatment algorithms for decentralised, primary-care led approach in Cambodia 5](#_Toc211263310)

# **National response to hepatitis C virus (HCV) in Cambodia**

Hepatitis C virus (HCV) was initially considered a low programmatic priority, with care and treatment primarily centralised at specialised clinics and national hospitals.^1^ Patients typically required multiple visits with specialists to complete the diagnostic and treatment course.^1^ The HCV screening and treatment programme began with Médecins Sans Frontières (MSF; 2016–2021) in collaboration with the Cambodian Ministry of Health (MoH), with the aim of screening and treating HCV for both HIV co-infection and mono-infection at the Hepato-Gastroenterology Department of the Cambodia–China Friendship Preah Kossamak Hospital (PKH), a government-operated national hospital in Phnom Penh.^2,3^ Between September, 2016 and June, 2019, 9158 viraemic patients initiated direct-acting antivirals (DAAs; Sofosbuvir and Daclatasvir), of whom 108 (1·2%) and six (0·1%) were co-infected with HIV and HIV/HBV, respectively.^1^ The study reported high treatment effectiveness, with up to 97·2% of patients achieving a sustained virological response at 12 weeks post-treatment (SVR12).

In 2018, MSF expanded the programme and initiated a trial of a decentralised HCV care model with simplified procedures, shifting key tasks such as HCV screening, diagnosis, patient assessment, and treatment initiation to non-specialists (nurses) at primary health care facilities in three operational districts in Battambang province.^4–6^ This model required fewer visits from screening to HCV viral load to achieve SVR12. Between 2018 and 2020, 21,385 individuals were screened, 1698 (7·9%) were anti-HCV-positive, and 1087 (5·0%) of all screened were viraemic.^4,5^ Among all viraemic patients, 1063 (97·8%) initiated DAA treatment, and 1040 (97·8%) completed the treatment course. The treatment effectiveness rate was high; ~97% of patients achieved SVR12 across studies.

Parallel to these pilot programmes, the National Centre for HIV/AIDS Dermatology and STD (NCHADS) established a national guideline for the management of individuals with HIV and HCV co-infection in 2017, with support from the Global Fund.^3^ The aim of the programme was to establish sustainable HCV care and treatment to ensure a cure for people living with HIV (PLHIV).^7,8^ As of 2021, ~80% of the antiretroviral therapy (ART) population had been screened for HCV.^7^ The current guideline recommends HCV screening for all PLHIV upon enrolment in ART services and facilities across Cambodia.^7^ When resources are limited, screening is prioritised for high-risk populations (e.g., people who inject drugs [PWID], men who have sex with men [MSM], and prisoners) and those with a history of HCV risk exposure or symptomatic liver disease.

In 2018, the National Viral Hepatitis Technical Working Group (TWG) was established and subsequently published a National Strategic Plan for Viral Hepatitis B and C Control in Cambodia (2020-2024), along with a plan for the national scale-up of the elimination programme.^2,9^ Building on the MSF pilot and the NCHADS HIV/HCV co-infection programme,^4,5,8^ the Communicable Disease Control Department of the MoH launched the National Viral Hepatitis Elimination Programme in November, 2023.^10^ This initiative was designed to ensure that preventive, diagnostic, and treatment services are both accessible and affordable, with the ultimate aim of eliminating HCV as a public health threat by 2030.^9^ This state-run programme adopted a decentralised, primary care–led approach, with task-shifting to primary health care workers for screenings, eligibility treatment assessment, and DAA initiation.^5,11^ Screening is offered to all adults, with prioritisation on high-risk populations, including adults aged ≥ 45 years, healthcare workers, PLHIV, PWID or have other parenteral drug exposures, and those with household exposure to a family member with HCV.^10^ Implementation currently spans 15 operational districts across six provinces in Cambodia.

An interrupted time-series before the 2024 scale-up is not feasible because no routine, country- or region-level monitoring captured the HCV cascade prior to 2024. We therefore (i) describe the pre-2024 model of care and its key differences, (ii) add a comparative table (pre-2024 versus 2024 decentralised model alongside the HIV/HCV co-infection programme), and (iii) present a proxy subgroup trend using routinely collected data from HIV clinics among people living with HIV (PLHIV) co-infected with HCV.

**Table S1:** Comparison of pre-2024 centralised versus 2024 decentralised model of hepatitis C (HCV) and HIV/HCV co-infection programme.

| **Domain** | **Pre-2024 (centralised)** | **2024 scale-up (decentralised)** | **HIV/HCV co-infection** |
| --- | --- | --- | --- |
| **Access points** | Mainly secondary/tertiary hospitals; limited sites | Primary care facilities plus referral hospitals (nationwide roll-out) | All ART sites leveraged for HCV screening among PLHIV |
| **Screening** | Opportunistic anti-HCV at higher-level facilities | Routine RDT screening integrated in primary care | All PLHIV before ART enrolment |
| **Confirmation** | RNA testing centralised; specimens shipped; multiple visits | Standardised pathways; organised sample transport; fewer visits | Testing performed through the NCHAID laboratory network |
| **Turnaround time** | Prolonged due to batching/transport and re-attendance | Shortened due to streamlined logistics and on-site initiation | Shortened through the NCHAID laboratory network |
| **Treatment availability** | DAAs at limited hospital clinics; not in primary care | Pangenotypic DAAs available closer to patients; nurse-led starts | Pangenotypic DAAs initiate at ART sites |
| **Data systems** | No routine, aggregated cascade monitoring | Electronic register & routine cascade indicators | Co-infection data embedded in existing HIV patient monitoring and laboratory system |
| **Patient costs/logistics** | Multiple visits, travel to cities | Fewer visits, local access | Leverage HIV sample transport and clinic visits to minimise extra trips |

Abbreviations: HCV, hepatitis C virus; RNA, ribonucleic acid; RDT, rapid diagnostic tests; DAAs, direct-acting antivirals; ART, antiretroviral therapy; PLHIV, people living with HIV; NCHAID, National Centre for HIV/AIDS Dermatology and STD.

Table 2 presents a proxy subgroup of PLHIV co-infected with HCV observed at ART clinics in 2024 and data extracted from quarterly reports published by NCHADS.^12–15^ Overall, 5280 PLHIV were screened for HCV in 2024, with volumes expanding over the quarterly reports. Of those screened, 301/5280 (5·7%) were anti-HCV-positive. Among those who underwent confirmatory PCR testing, 186/446 (41·7%) had detectable HCV RNA, and treatment initiation exceeded ~86% in each quarter. Non-concordance between counts of anti-HCV tests, RNA testing/positivity, and treatment reflects earlier backlogs rather than over-enrolment in each step of the cascade. Overall, the pattern highlights maturing services with improved case-finding embedded in the ART services; nonetheless, these clinic-based data are not representative of the general population and should be interpreted as contextual to the HCV programme among PLHIV.

**Table S2:** Proxy subgroup trends among people living with HIV (PLHIV) in HIV clinics in 2024.

| **Period** | **HCV Screened (N)** | **Anti-HCV-positive**  **n/N (%)** | **RNA-positive**  **n/N (%)^a^** | **Initiated treatment**  **n/N (%)^b^** |
| --- | --- | --- | --- | --- |
| 2024-Q1 | 655 | 19/655 (2·9) | 20/42 (47·6) | 18/20 (90·0) |
| 2024-Q2 | 1905 | 87/1905 (4·6) | 45/148 (30·4) | 39/45 (86·7) |
| 2024-Q3 | 1014 | 75/1014 (7·4) | 49/98 (50·0) | 71/49 (144·9) |
| 2024-Q4 | 1706 | 120/1706 (7·0) | 72/158 (45·6) | 67/72 (93·1) |
| Total | 5280 | 301/5280 (5·7) | 186/446 (41·7) | 195/186 (104·8) |

Data are presented as n/N (%).

^a^ RNA-positive cases were calculated by dividing the number of RNA-positive individuals (n) by the total number of confirmatory tests performed (N). The programme frequently initiated backlogs testing, resulting in discrepancies between the number of confirmatory tests and the number of anti-HCV-positive screenings reported quarterly.

^b^ Initiated treatment was calculated by dividing the number of RNA-positive individuals who received direct-acting antivirals (DAAs) within a given quarter by the number of RNA-positive cases reported in that same quarter. The proportion may exceed 100%, reflecting cross-quarter initiation and does not imply over-enrolment.

# **Data Source**

Routine data from the National Viral Hepatitis Elimination Programme were obtained from the Communicable Disease Control Department (CDC) of the MoH. These data, collected routinely, were documented in the HCV logbooks at the health facility-level. On a monthly basis, health facilities reported key indicators directly to the CDC. These indicators included (1) point-of-care anti-HCV rapid diagnostic testing using WHO-prequalified assays at health centres and referral hospitals; (2) the number of individuals undergoing confirmatory HCV RNA testing (PCR) at designated laboratories; (3) the number of individuals with HCV RNA-positive who initiated pan-genotypic direct-acting antivirals (DAAs) at health facilities; (4) the number of RNA-positive patients who completed the full course of therapy; and (5) the number of patients lost to follow-up for both RNA testing and treatment. These indicators were disaggregated by age group (≤45 years and >45 years), sex (male and female), and time of reporting (calendar month).

The population denominator for national HCV testing coverage was obtained from operational health district, which was based on Cambodia Population Projection 2020-2023 (National Institute of Statistics, Ministry of Planning), derived from the General Population Census 2019, and the Cambodia Health Demographic Survey 2021-2022.^16^

# **Data Storage and Security**

The dataset comprised de-identified, aggregated health facility-level data. Data were stored and managed at CDC, and only authorised persons had access to the dataset.

# **Data Quality Procedures**

To enhance the data quality of the programme, routine checks were implemented at three levels:

1. Indicator dictionary: All variables reported from health facilities were defined in an indicator dictionary, including denominators and disaggregation by age group (≤45 years versus >45 years), sex (male versus female), and monthly reporting period. The dictionary served to enhance and standardise indicators across health facilities and reporting periods.
2. Range checks: Quantitative data were verified and checked across indicators to ensure consistency and improve data quality. These checks included the following:
3. The number of HCV rapid diagnostic tests verified against the number of anti-HCV-positive cases, by age group, sex, health facility, and on a monthly basis.
4. The number of RNA tests (PCR) cross-checked against the number of anti-HCV-positive cases, by age group, sex, health facility, and on a monthly basis.
5. The number of patients initiating DAA cross-checked against the number of RNA-positive cases, by age group, sex, health facility, and on a monthly basis.
6. The number of patients completing DAA treatment cross-checked against the number of RNA-positive patients, by age group, sex, health facility, and on a monthly basis.
7. The number of patients lost to follow-up cross-checked against the number of anti-HCV-positive cases, RNA testing, RNA-positive cases, and DAA therapy, by age group, sex, health facility, and on a monthly basis.
8. Reconciliation with laboratory tallies: health facility reports RNA-testing and RNA-positive were compared with referral/provincial laboratory registers.

# **Data Exclusion Criteria**

Our study included all individuals accessing HCV services at participating facilities between 1 January and 31 December 2024. Monthly health facility-level data were excluded if they did not meet predefined criteria and data completeness requirements:

- Patients aged <18 years were excluded from data analysis.
- Patients who were tested for HCV and initiated DAA before 1 January 2024 were excluded.
- At least 95% of the required indicators and variables must be presented.
- At least 95% agreement with laboratory tally reports was required.
- Health facilities that missed more than three consecutive months of reporting were excluded from the time-series analysis.

# **Data Cleaning Procedure**

Step 1: Raw data were obtained from CDC and imported in Excel format.

Step 2: As the data were reported in the Khmer language, they were transformed into English and then merged into a single Excel file. This process was conducted using the R programme.

Step 3: Variables and indicators were standardised based on the data dictionary.

Step 4: An R script was developed and utilized for range and consistency checking.

Step 5: Data-quality reports were generated by health facility month. Data inconsistencies and out-of-range values were verified with the health facilities to ensure accuracy compared with the physical logbooks stored at the site.

Step 6: All exclusion criteria were applied to generate a final clean dataset for analysis.

# **Documentation**

Data discrepancies detected through routine data checks, verification, reconciliation with laboratory reports, or inconsistencies in monthly submissions were systematically documented and communicated to health facilities and the CDC. Then these discrepancies and inconsistencies were cross-checked and verified against physical logbooks or laboratory registers at each health facility. Once confirmation of errors, corrected data was resubmitted and incorporated into the central database at CDC. Any manual corrections were logged with the date, reason, and initials of the reviewer and responders.

**Figure S1:** Hepatitis C virus (HCV) testing and treatment algorithms for decentralised, primary-care led approach in Cambodia


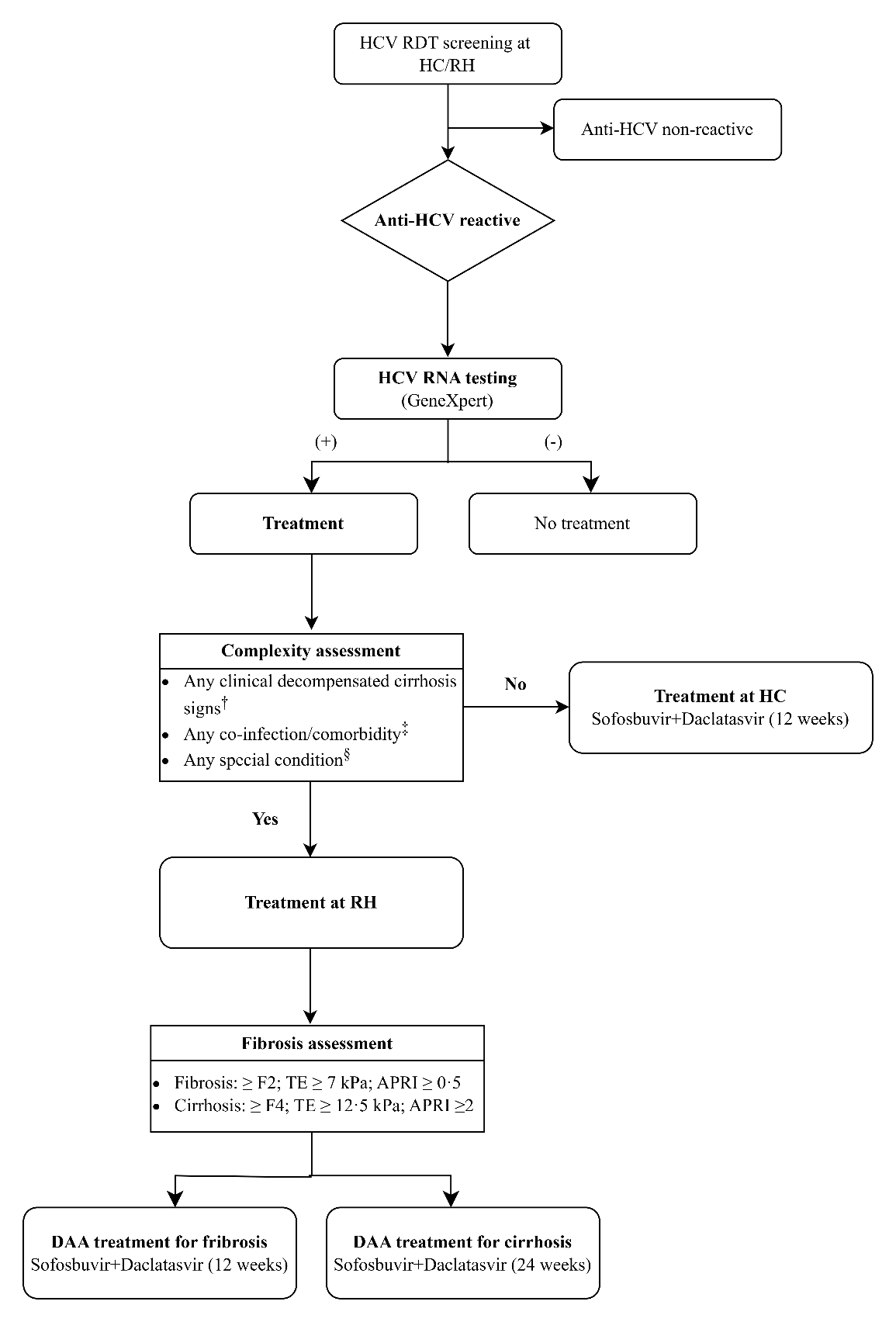


Abbreviations: HCV, hepatitis C virus; RDT, rapid diagnostic test; HC, health centre; RH, referral hospital; (+), detectable HCV RNA;

(-), undetectable HCV RNA; F, FibroScan stage; TE, transient elastography, APRI, aminotransferase/platelet ratio index; kPa, kilopascal

† Clinical cirrhosis: pruritus, fatigue, arthralgia, palmar erythema, oedema, jaundice, hepatomegaly, ascites, variceal haemorrhage, and hepatic encephalopathy.

‡ Co-infection: any co-infection (HBV, HIV, or TB); Comorbidity: diabetes, or higher blood pressures, etc.

§ Special condition: people who inject drugs, individuals with mental health, pregnant women, and breastfeeding women.

# **References**

1. Zhang M, O’Keefe D, Iwamoto M, Sann K, Kien A, Hang V, et al. High sustained viral response rate in patients with hepatitis C using generic sofosbuvir and daclatasvir in Phnom Penh, Cambodia. J Viral Hepat. 2020 Sept;27(9):886–95.

2. Hugues J, Sharon M, Glenn O. Lessons Learnt: The MSF governance model of the UNITAID Grant “Ensuring access to the HCV treatment - revolution for HCV/HIV co-infected patients in LMICs” [Internet]. Vienna: TRAASS International; Médecins Sans Frontières Vienna Evaluation Unit; 2018 Oct. Available from: https://evaluation.msf.org/sites/default/files/attachments/unitaid_hcv_eval_report_final_openaccess.pdf

3. Dalberg. Ensuring access to the Hepatitis C (HCV) treatment revolution for HCV/HIV co-infected patients in LMICs [Internet]. Unitaid; 2019 Feb. Available from: https://unitaid.org/uploads/Ensuring-access-to-the-Hepatitis-C-HCV-treatment-revolution-for-HCV-HIV-co-infected-patients-in-LMICs.pdf

4. Zhang M, O’Keefe D, Craig J, Samley K, Bunreth V, Jolivet P, et al. Decentralised hepatitis C testing and treatment in rural Cambodia: evaluation of a simplified service model integrated in an existing public health system. Lancet Gastroenterol Hepatol. 2021 May;6(5):371–80.

5. O’Keefe D, Samley K, Bunreth V, Marquardt T, Bobi SE, Antharo K, et al. Nurse-led initiation of hepatitis C care in rural Cambodia. Bull World Health Organ. 2023 Apr 1;101(04):262–70.

6. Taithe B, le Paih M. Making Hepatitis C History? Médecins sans Frontières, Hepatitis C and Humanitarian Medicine in Cambodia 2016–20211. Soc Hist Med. 2025 June 20;hkaf040.

7. National Centre for HIV/AIDS Dermatology and STDs. Strategic Plan for HIV and STI Prevention and Care in the Health Sector 2021-2025 in Cambodia [Internet]. 2021 [cited 2025 Sept 23]. Available from: https://www.nchads.org/wp-content/uploads/2021/06/SPHIV-STIs-2021-2025-1.pdf?

8. National Centre for HIV/AIDS Dermatology and STDs. National guidelines for management of persons with HIV and hepatitis C co-infection [Internet]. 2017. Available from: https://www.nchads.org/wp-content/uploads/2021/02/National-Guidelines-for-Management-of-Persons-with-HIV-HCV-CoInfections-Eng.pdf

9. Communicable Diseases Control Department, Ministry of Health Cambodia. National Strategic Plan for Viral Hepatitis B and C Infection Control in Cambodia 2020-2024 [Internet]. Ministry of Health, Cambodia; 2019. Available from: http://cdcmoh.gov.kh/surveillance/viral-hepatitis

10. Communicable Diseases Control Department, Ministry of Health Cambodia. Guideline of Clinical Management for Viral hepatitis C [Internet]. Communicable Diseases Control Department, Ministry of Health Cambodia; 2019. Available from: http://cdcmoh.gov.kh/images/Document/Viral_Hepatitis/NSP-VH%E2%80%8B_final191220_Eng.pdf

11. World Health Organization. Updated recommendations on simplified service delivery and diagnostics for hepatitis C infection: Policy Brief. 1st ed. Geneva: World Health Organization; 2022. 1 p.

12. National Centre for HIV/AIDS Dermatology and STDs. Quarter 1-2024: HIV/AIDS Treatment and HCV-HIV Co-infection Report [Internet]. Phnom Penh; 2024 Apr [cited 2025 Oct 2]. Available from: https://www.nchads.gov.kh/wp-content/uploads/2024/04/ART-National-Report-Q1_2024.pdf

13. National Centre for HIV/AIDS Dermatology and STDs. Quarter 2-2024: HIV/AIDS Treatment and HCV-HIV Co-infection Report [Internet]. Phnom Penh; 2024 July [cited 2025 Oct 2]. Available from: https://www.nchads.gov.kh/wp-content/uploads/2024/07/Report-on-ART_Q2_2024.pdf

14. National Centre for HIV/AIDS Dermatology and STDs. Quarter 3-2024: HIV/AIDS Treatment and HCV-HIV Co-infection Report [Internet]. Phnom Penh; 2024 Oct [cited 2025 Oct 2]. Available from: https://www.nchads.gov.kh/wp-content/uploads/2024/10/National-Report-on-ART_Q3_2024.pdf

15. National Centre for HIV/AIDS Dermatology and STDs. Quarter 4-2024: HIV/AIDS Treatment and HCV-HIV Co-infection Report [Internet]. Phnom Penh; 2025 Jan [cited 2025 Oct 2]. Available from: https://www.nchads.gov.kh/wp-content/uploads/2025/01/Final_National-Report_Q4_2024.pdf

16. National Institute of Statistics of Ministry of Planning. Population Projection 2020-2033 Based on Age Restructure of National Population Projection 2019-2050 and CDHS 2021-2 [Internet]. National Institute of Statistics; 2023. Available from: https://www.nis.gov.kh/nis/Population%20Projection/Cambodia_Population_Projection_2020-2033.pdf
